# Designing for delivery: a Delphi study of feasibility-informed implementation strategies for a fear of cancer recurrence clinical pathway

**DOI:** 10.1007/s00520-026-11275-9

**Published:** 2026-10-09

**Authors:** Ben Smith, Verena Shuwen Wu, Alison Pearce, Jia Liu, Heather L. Shepherd, Joanne Shaw, Afaf Girgis, Gail Garvey, Laura Kirsten, Iman Zakhary, Carolyn Ee, Dan Ewald, Annie Miller, Andrea Smith, Agamjot Kaur Virk, Joy Gao, Natalie Taylor

**Affiliations:** 1 The Daffodil Centre, The University of Sydney, and Cancer Council NSW, Sydney, Australia; 2 South West Sydney Clinical Campuses, UNSW Medicine & Health, UNSW Sydney, Sydney, Australia; 3 Sydney School of Public Health, The University of Sydney, Camperdown, Australia; 4 The Kinghorn Cancer Centre, St Vincent’s Hospital, Sydney, NSW Australia; 5 St Vincent’s Clinical School, UNSW Sydney, Darlinghurst, NSW Australia; 6 Susan Wakil School of Nursing and Midwifery, Faculty of Medicine and Health, The University of Sydney, Camperdown, NSW Australia; 7 Psycho-Oncology Cooperative Research Group, The University of Sydney, Camperdown, NSW Australia; 8 School of Psychology, Faculty of Science, The University of Sydney, Camperdown, Australia; 9 School of Public Health, The University of Queensland, Brisbane, Australia; 10 Nepean Cancer Services, Nepean Blue Mountains Local Health District, Kingswood, Australia; 11 Multicultural Services, Liverpool Hospital, South Western Sydney Local Health District, Sydney, Australia; 12 Flinders LiveWell Cancer Research Centre, Caring Futures Institute, Flinders University, Adelaide, Australia; 13 NICM Health Research Institute, Western Sydney University, Sydney, NSW Australia; 14 Chris O’Brien Lifehouse Cancer Centre, Camperdown, NSW Australia; 15 Sydney University Medical School, Northern Rivers University Centre for Rural Health, Lismore, NSW Australia; 16 Implementation to Impact (i2i), School of Population Health, UNSW Sydney, Sydney, Australia

**Keywords:** Fear of cancer recurrence, Cancer survivorship, Clinical pathway, Supportive care, Implementation, Delphi study

## Abstract

**Purpose:**

Fear of cancer recurrence (FCR) is pervasive among cancer survivors, yet evidence-based care is rarely implemented. Clinical pathways offer a structured approach to translating evidence into practice. This study identified key implementation barriers, enablers, and strategies for feasibly integrating an FCR clinical pathway comprising screening, assessment, and triage to stepped care into practice.

**Methods:**

A three-round Delphi study was conducted with Australian health professionals and FCR researchers. Participants rated and provided qualitative feedback on the feasibility of FCR clinical pathway elements. Qualitative responses were content analysed using the updated Consolidated Framework for Implementation Research. Recommended strategies were mapped using the Expert Recommendations for Implementing Change taxonomy.

**Results:**

Eighty-nine participants completed Round 1, 69 Round 2, and 73 Round 3. Feasibility ratings varied across pathway elements (49–90%). Stepped care elements were widely endorsed as feasible (71-83% agreement), while resource-intensive recommendations (e.g., training all staff for screening) were perceived as least feasible. Key implementation enablers included Adaptability, Access to Knowledge and Information, and strong Innovation Recipient Need. Major barriers were Available Resources, Work Infrastructure and limited Innovation Deliverer Opportunity. Recommended strategies included tailoring delivery to local contexts and patient needs supported by modular training and local champions.

**Conclusion:**

Implementing the FCR clinical pathway requires pragmatic adaptation and system-level support. Prioritising high-impact elements, providing training, integration into existing workflows, and shared decision-making to meet patient needs are critical. Future research should evaluate these strategies using hybrid effectiveness–implementation designs to ensure sustainable implementation and improved patient outcomes.

**Supplementary Information:**

The online version contains supplementary material available at https://doi.org/10.1007/s00520-026-11275-9.

## Introduction

The number of people living with and beyond cancer is growing rapidly, currently estimated to be over 53 million people globally [1]. Most people affected by cancer experience some degree of fear, worry, or concern about their cancer coming back or getting worse, which is referred to as fear of cancer recurrence (FCR) [2]. An international individual participant data meta-analysis (*n* = 9311) found 39% of people affected by cancer experience moderate FCR, while 19% experience severe/clinical FCR [3] equating to more than 30 million people affected by FCR globally. People with clinically severe FCR experience high and persistent levels of preoccupation and worry, hypervigilance to physical symptoms, and functional impairment [4]. Moreover, people with more severe FCR experience poorer mental health and quality of life [5], and use more healthcare services [6].

Receiving help with FCR has been identified as the greatest unmet supportive care need in people with cancer [7]. In response, a wide range of measures (e.g. the Fear of Cancer Recurrence Inventory [8], FCR-4/7 [9], Ottawa Clinical Fear of Recurrence instruments [10]) and treatments (e.g. ConquerFear [11], FORT [12], SWORD [13]) have been developed and shown to effectively identify, assess, and treat FCR [14, 15]. However, translating these resources into routine clinical practice has proven challenging. Documented implementation barriers include limited awareness of FCR, inadequate referral pathways, concerns about equitable access, healthcare professional capacity constraints, and challenges applying manualised FCR interventions in diverse clinical contexts [16, 17]. Until recently, there was limited formal guidance on how to systematically address FCR in clinical settings. In 2024, this gap was addressed through the publication of Canadian FCR guidelines [18] and a complementary Australian FCR clinical pathway developed by our team [19]. Both advocate for routine FCR screening, followed by further assessment and triage to stepped care. Pilot studies have demonstrated the acceptability, feasibility, and preliminary efficacy of this approach [20], but there is limited evidence of successful implementation in routine care. Enhancing the translation of FCR research into clinical care and improving access to FCR treatment have been identified as international [21] research priorities.

Clinical pathways are a well-established strategy for translating evidence into practice. By offering standardised, context-specific recommendations for multidisciplinary care, they help bridge the gap between research and routine clinical delivery [22]. To support implementation of FCR research, we developed a clinical pathway incorporating contemporary evidence and consensus-based guidance on optimal FCR screening, assessment, and triage to stepped care [19]. The pathway was developed through a three-stage Delphi process, whereby *n* = 94 participants—primarily health professionals working across varied disciplines and settings—reached consensus on 35/38 (92%) of proposed pathway elements based on alignment with best-practice and potential to improve patient outcomes (optimality). With consensus achieved regarding the optimal clinical pathway, the next critical step is to ensure feasibility of pathway elements. Feasibility was also assessed in the Delphi study and has been identified as a key antecedent of successful implementation. Analysis of qualitative feedback regarding feasibility can identify strategies to overcome perceived implementation barriers and enablers.

This secondary analysis of data from the Delphi study conducted to establish the optimal FCR clinical pathway aimed to facilitate the successful future implementation of the pathway by:

1. Reporting the perceived feasibility of optimal pathway elements.
2. Identifying pathway implementation barriers and enablers.
3. Developing pathway implementation strategies.

## Methods

### Study design and context

This study is a secondary analysis of data collected during a previously published three-round Delphi study that established consensus on optimal elements of a clinical pathway for identifying and managing FCR in routine care of early-stage cancer survivors [19].

The study was approved by the University of New South Wales Human Research Ethics Committee (HC230052), conducted in accordance with the Conducting and Reporting Delphi Studies (CREDES) guidelines [23], and reported following the Standards for Reporting Implementation Studies (StaRI) Statement [24].

### Participants and recruitment

Eligible participants included Australian health professionals involved in the care of adult cancer survivors (≥ 18 years) and researchers who had co-authored a peer-reviewed paper on FCR within the past 5 years. Participants working primarily with paediatric populations were excluded due to contextual differences in FCR presentation and management. Participants were recruited via professional oncology organisations, conference presentations, and social media. All participants gave informed consent. Participants who completed Round 1 were invited to subsequent rounds; no new participants were recruited after Round 1.

### Outcomes and analysis

#### Feasibility assessment (Aim 1)

Participants rated the feasibility (i.e. likelihood of implementation in routine care) of each pathway element using a 5-point Likert scale from “strongly disagree” to “strongly agre,”, with a “Not my expertise” option for items outside a participant’s scope.

The proportion of participants agreeing/strongly agreeing that an item was feasible was analysed descriptively.

#### Identifying pathway implementation barriers and enablers (Aim 2)

Open-ended comments were collected alongside the Delphi item for each pathway element and at the end of each pathway component to capture perceived implementation barriers and enablers.

Directed content analysis was conducted on open-ended qualitative responses using the updated Consolidated Framework for Implementation Research (CFIR) [25]. Only responses related to pathway elements included in the final clinical pathway were analysed. The updated CFIR is one of the most widely used and validated frameworks for categorising implementation barriers and enablers (i.e. determinants). It comprises five domains: Innovation (intervention attributes), Outer Setting (external context), Inner Setting (organisational context), Individuals (characteristics of those involved in implementation), and Implementation Process (implementation activities) [25]. Each domain includes multiple constructs representing potential barriers and enablers to implementation. As the clinical pathway is currently in the pre-implementation phase, the Implementation Process domain was not coded.

A codebook was developed by the research team based on the updated CFIR template (available at cfirguide.org) and adapted to the study context to aid consistent interpretation of constructs and support inter-rater reliability (see Supplementary Material 1). Each qualitative response was independently coded by two researchers (AKV and JG) to the single most relevant CFIR domain and construct. Coders also classified whether the response reflected an implementation barrier, enabler, or neutral factor. The number of barriers and enablers relating to each construct was counted.

Coding followed an iterative process. AKV and JG met regularly with the broader research team to discuss and refine coding decisions. Discrepancies were resolved through team consensus involving experienced qualitative/implementation researchers (ABS, AS, and VW). To enhance reliability, ABS and VW subsequently each coded half of the dataset, so every response was reviewed by three researchers. All coding was conducted using Microsoft Excel.

#### Developing implementation strategies (Aim 3)

Once key implementation determinants were identified, the CFIR-ERIC matching tool [26] was used to identify implementation strategies addressing the most common barriers and enablers. The CFIR-ERIC matching tool is a theory-informed resource that links specific implementation barriers and enablers identified using the CFIR, to evidence-based implementation strategies from the Expert Recommendations for Implementing Change (ERIC) taxonomy [27]. It enables systematic selection of strategies most likely to address identified contextual challenges, thereby enhancing the feasibility and likely success of implementation.

## Results

### Participants

Of the 94 individuals who consented to participate in the Delphi study, 89 completed Round 1 (April–June 2023), 69 completed Round 2 (July–August 2023), and 73 completed Round 3 (November–December 2023). Participants (see Table 1) were primarily healthcare professionals (86%), spanning disciplines including mental and allied health (*n* = 31, 33%), nursing (*n* = 30, 32%), and medical/radiation/surgical oncology (*n* = 18, 19%) working in metropolitan (84%) tertiary referral centres (47%) in public settings (54%).

**Table 1:** Participant characteristics

Characteristic		*N* (%) of respondents
Age	21–30	3 (3.2)
	31–40	25 (26.6)
	41–50	28 (29.8)
	51–60	24 (25.5)
	61–70	13 (13.8)
	Prefer not to say	1 (1.1)
Gender	Man/male	10 (10.6)
	Woman/female	84 (89.4)
Country of birth	Australia	71 (75.5)
	Other	23 (24.5)
Language spoken at home	English	82 (87.2)
	Other	12 (12.8)
Aboriginal/Torres Strait Islander status	Non-Indigenous	94 (100.0)
Geographic area	Metropolitan	79 (84.0)
	Regional	5 (5.3)
	Rural	10 (10.6)
Primary role	Clinician	81 (86.2)
	Researcher	10 (10.6)
	Educator	3 (3.2)
Primary focus	Psychology	18 (19.1)
	Social work	13 (13.8)
	Nursing	30 (31.9)
	Medical oncology	12 (12.8)
	Radiation oncology	5 (5.3)
	Surgical oncology	1 (1.1)
	Palliative care	1 (1.1)
	Other	14 (14.9)
Work setting (clinician/educator; *n* = 84)^a^	Tertiary referral cancer centre	44 (46.8)
	District/local hospital	22 (23.4)
	Non inpatient cancer treatment centre	18 (19.1)
	Non-hospital based	10 (10.6)
	Primary care	2 (2.1)
	Community centre	6 (6.4)
	Other	6 (6.4)
Public/private (clinician/educator; *n* = 84)^a^	Public	51 (54.3)
	Private	7 (7.4)
	Public and private	17 (18.1)
	Public charitable organisation	3 (3.2)
	Private charitable organisation	5 (5.3)
	Other	2 (2.1)
Years worked with cancer survivors (clinician/educator; *n* = 84)	0–2	3 (3.2)
	3–5	11 (11.7)
	6–10	22 (23.4)
	10 +	48 (51.1)
Role caring for cancer survivors with FCR (clinician/educator; *n* = 84)^a^	Screening and/or assessment	49 (52.1)
	Referral to psychosocial support	55 (58.5)
	Provision of psychosocial support	47 (50.0)
Years conducting research (researcher; *n* = 10)	6–10	3 (3.2)
	10 +	7 (7.4)

^a^Respondents could select multiple responses

### Feasibility of pathway elements (Aim 1)

Feasibility ratings for clinical pathway elements ranged from 49.4% (“All clinical staff should be trained to implement FCR screening and respond appropriately”) to 90.1% (“It is important that all health care professionals validate and normalise FCR”) (see Table 2). Average feasibility ratings were highest for the post-screening/triage stepped-care treatment pathway component (75.9%) and lowest for the supported self-management for moderate FCR pathway component (61.0%).

**Table 2:**
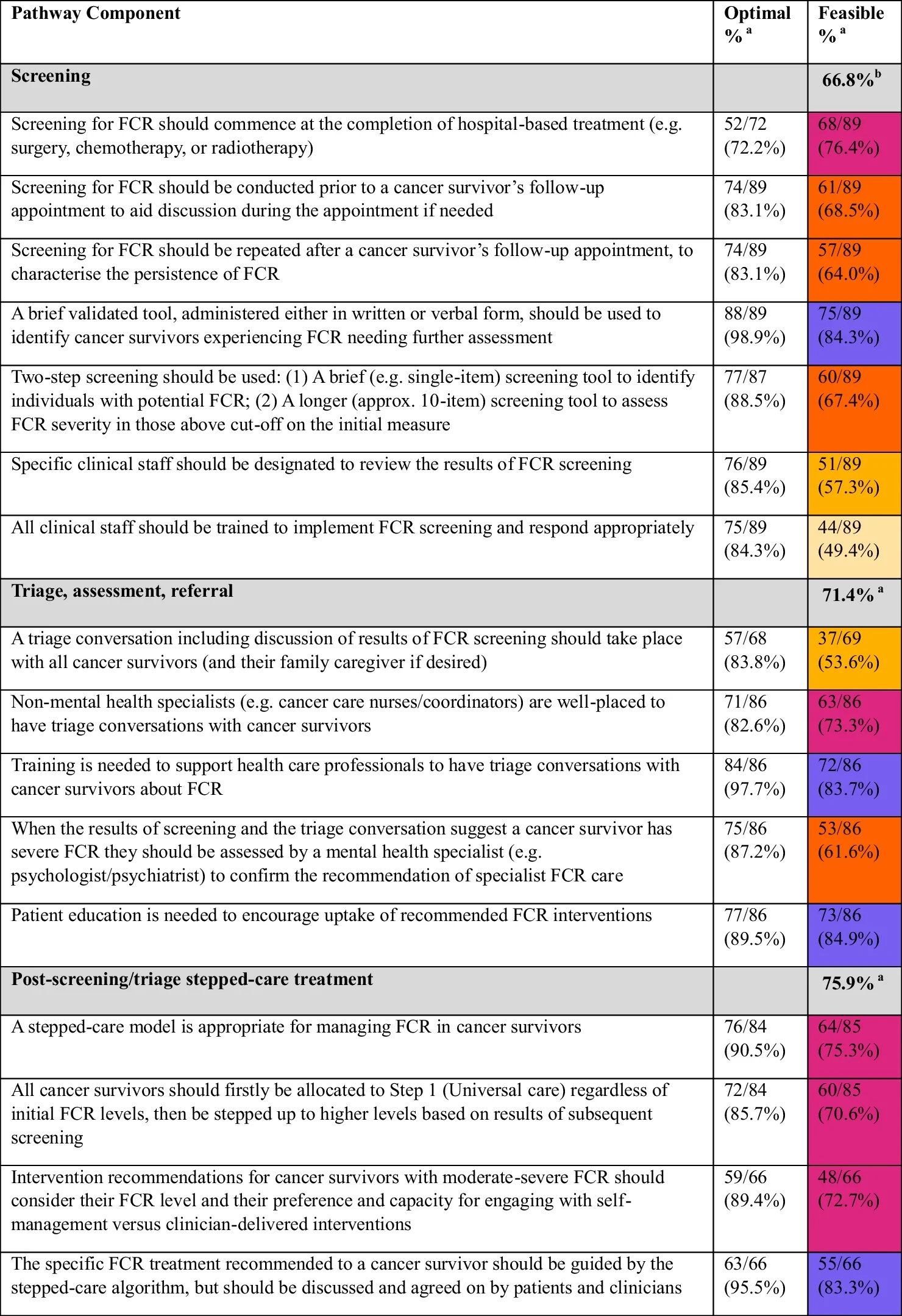

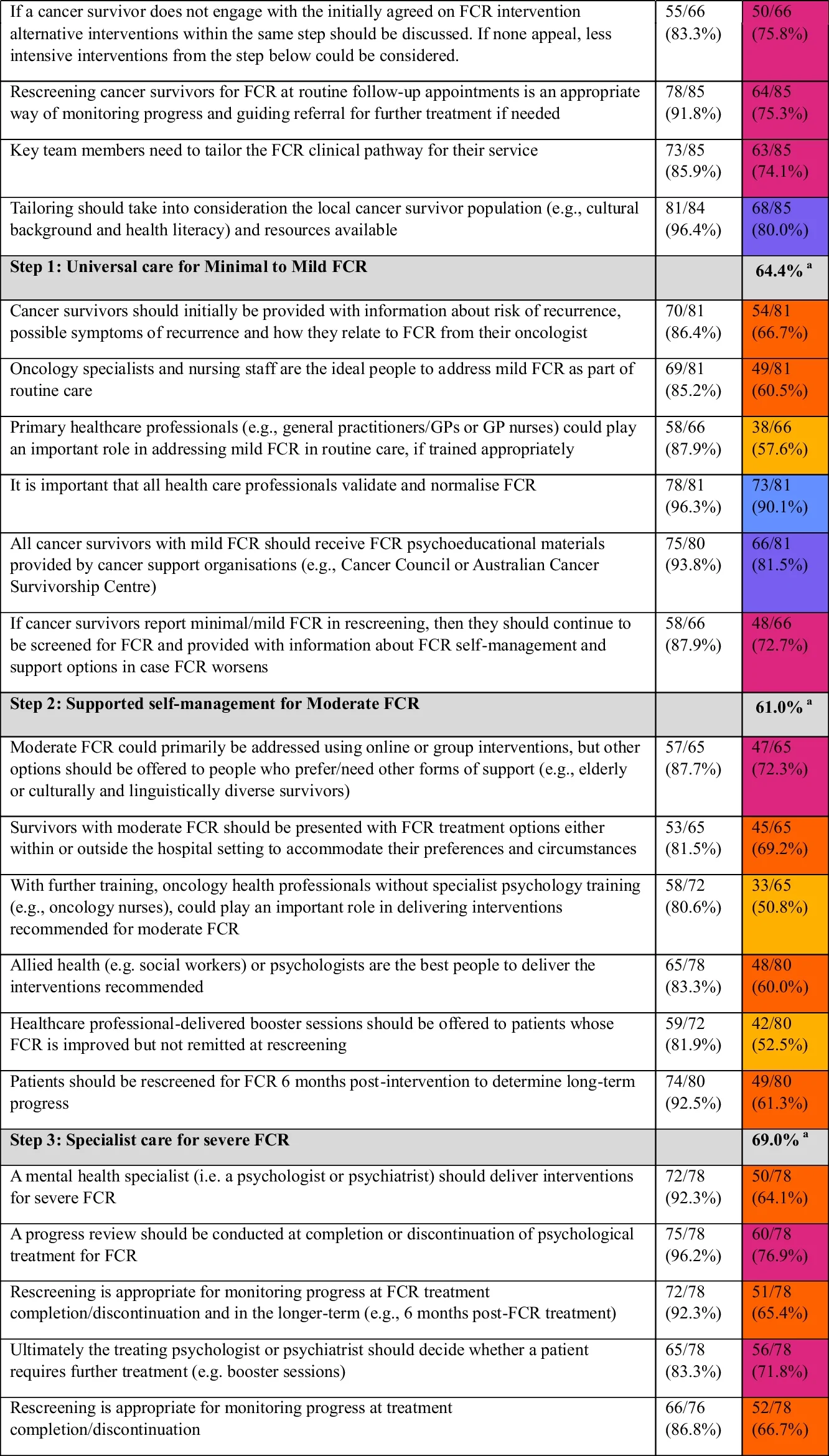
Optimal and feasible ratings for elements included in the FCR clinical pathway

^a^Reported percentages represent the number of participants who agreed or strongly agreed with the item divided by the number of people who responded to that item, excluding those who indicated “Not my expertise”. The highest optimal rating was taken from across rounds, and items were rated for feasibility once across all rounds, which is why denominators differ for the optimality and feasibility ratings within items and feasibility ratings across items

^b^Average feasibility for each pathway component was calculated by summing the percentage for each individual element included in the pathway and dividing by the total number of included elements in that component

*Shading denotes strength of feasibility ratings. Blue indicates 90–100% agreement; purple indicates 80–90% agreement; pink indicates 70–80% agreement; orange indicates 60–70% agreement; dark yellow indicates 50–60% agreement; and light yellow indicates < 50% agreement

### Determinants of pathway feasibility (Aim 2)

Of the 639 qualitative responses collected, 410 related to elements ultimately included in the FCR clinical pathway, which were coded to the Innovation (*n* = 99), Individuals (*n* = 125), Inner Setting (*n* = 144), and Outer Setting (*n* = 9) updated CFIR domains. Thirty-three responses were coded as ‘not applicable’ due to brevity or ambiguity.

170 responses were coded as barriers, 53 as neutral, and 154 as enablers. Barriers and enablers related to updated CFIR domains (in bold italics) and constructs (in bold) are provided below, with illustrative quotes in Table 3.

**Table 3:** Most common implementation barriers and enablers according to updated CFIR domains and constructs

CFIR domain	Construct	Type	Coding frequency	Illustrative quotes
Innovation	Innovation Design	Enabler	20	This [FCR triage conversations with all survivors and caregivers] is consistent with best practice
	Innovation Design	Barrier	8	Screening in the immediate window prior to a follow-up appointment may pick up a lot of transient peaks (not the persistent/disabling FCR)
	Innovation Complexity	Barrier	7	[Two-step screening] increases administration burden and reduces feasibility While simple/straightforward, long term this [stepped care] impacts feasibility, as it will lead to increased “double handling” (i.e. for the ~ 60% of patients that will need to be stepped up)
	Innovation Adaptability	Enabler	22	A variety of means for administering the [FCR screening] tool should be used, especially for rural & remote patients “[FCR treatment] may need to be tailored around geographical location and availability of services.”
Individuals	Innovation Deliverer Capability	Enabler	19	Any clinician could administer the [brief FCR screening] tool—novice to expert Social workers and mental health occupational therapists are well positioned to address FCR
	Innovation Recipient Need	Enabler	25	[FCR screening] Can assist patients identify and name their emotions and give permission to talk Important to be patient-centred and facilitate their autonomy of choice
	Innovation Deliverer Opportunity	Barrier	15	Not all clinical staff have the time or capacity to do this [FCR screening] in their clinic Accessing [allied health professionals/psychologists] within a timely manner [is] often difficult due to workload demands
	Innovation Recipient Need	Barrier	15	Joint decision with the patient and psychologist [regarding further treatment for severe FCR]
	Innovation Deliverer Opportunity	Enabler	11	Would be feasible if the screening was initiated by other clinical staff (e.g. clinical trials or care coordinators)
Inner Setting	Access to Knowledge and Information	Enabler	21	Education of all staff will increase uptake Online/module-based training (e.g. via EviQ) may be sufficient
	Access to Knowledge and Information	Barrier	7	Maintaining training to ensure all staff are upskilled is a challenge
	Work Infrastructure	Barrier	30	[Assessment or treatment for severe FCR is] dependent on whether a service has a mental health specialist on staff We barely cover significantly distressed active treatment patients, who have to take priority
	Work Infrastructure	Enabler	35	Always more nurses than psycho-oncology staff
	Available Resources	Barrier	31	Most social workers or psychologists would be unable to undertake this work unless additional hours were funded for that purpose Not all rural and remote have access to either [allied health/psychology]
	Structural Characteristics	Barrier	15	If there is really only one service, this [two-step screening] is likely a waste of time
	Structural Characteristics	Enabler	4	Staff go on leave etc., so needs widespread ownership
	Compatibility	Enabler	6	[FCR triage conversations are feasible] If implemented as routine screening practice within consultation
	Compatibility	Barrier	3	Administratively, this [6 month post-intervention rescreening] would be very difficult to determine
Outer Setting	Partnerships and Connections	Enabler	3	FCR interventions can be well delivered outside the treatment environment… these can be funded through other streams – Medicare, Private Health
	Local Conditions	Barrier	6	In the community setting there can be barriers to accessing psychological support; wait times for appointments, lack of oncology experienced psychology services in the area, costs associated with this Culturally and linguistically diverse survivors, especially elderly people, don't like to talk about mental health

#### Innovation domain

Forty-five enablers, 22 barriers, and 32 neutral factors were identified regarding *Innovation*, namely the FCR clinical pathway.

*Innovation design* was both an enabler (*n* = 20) and a barrier (*n* = 8). Many participants viewed the pathway’s design as aligned with best practice*,* but some thought screening prior to follow-up appointments may pick up transient peaks in FCR.

*Innovation complexity* was identified as a barrier (*n* = 7). The two-step screening process was seen as adding administrative burden and concerns were also raised about stepped care leading to ‘double handling’ of the majority of patients likely to need extra support.

*Innovation adaptability* was the most frequently cited enabler (*n* = 22). Participants emphasised the importance of tailoring implementation to local contexts, especially for rural and remote patients with limited access to in-person care and psychological support. This flexibility was seen as critical to supporting implementation across diverse settings and populations.

#### Individuals domain

Sixty-two enablers, 51 barriers, and 12 neutral factors were identified regarding *Individuals*, namely the people involved in pathway implementation.

*Innovation deliverer capability* was a key enabler (*n* = 19). Participants generally viewed FCR screening as within the *capabilities* of most staff. The *capability* of allied health professionals to address FCR across severity levels was also highlighted. Building *capability* through training was considered essential for some pathway elements, such as triage conversations and normalising FCR.

Responsiveness of the pathway to *innovation recipient need* was a strong enabler (*n* = 25). Screening was seen as empowering patients to discuss FCR. Participants emphasised the importance of supporting patient-centred care by enabling intervention choice within a stepped care model. This construct was also considered a barrier (*n* = 15) when pathway recommendations were perceived as misaligned with recipient preferences or capacity. For example, some felt oncologists should only provide recurrence risk information if patients wanted it. Similarly, recommendations that treating psychologists/psychiatrists should decide whether further treatment for severe FCR was warranted prompted calls for shared decision-making.

Views were mixed regarding *innovation deliverer opportunity*. Barriers (*n* = 15) included time and workload constraints limiting screening opportunities for some clinical staff*.* Integrating FCR triage into routine follow-up was seen as challenging due to the competing demands of medical management. Limited opportunity to provide FCR-related support was noted across roles, with oncologists particularly constrained. While allied health professionals and psychologists were viewed as best suited for moderate FCR interventions, access was limited. Enablers (*n* = 11) included training non-mental health specialists (e.g. nurses), though workload pressures remained a concern. Expanding access through community or private providers was also proposed.

#### Inner setting domain

Forty-five enablers, 91 barriers, and 8 neutral were identified regarding the *Inner Setting*, namely the organisational context of clinical pathway implementation.

*Access to knowledge and information* was the most frequently cited enabler (*n* = 21). Participants emphasised training and education as critical for pathway uptake. It was noted that modular online training may be sufficient to support healthcare professionals to have triage conversations. Training was viewed as essential for validating and normalising FCR and that training non-mental health specialists (e.g. nurses) to deliver interventions for moderate FCR could extend pathway reach. Mental health specialists were considered to have a fundamental knowledge base regarding FCR treatment but require continuing professional development. Barriers (*n* = 7) included time constraints and staff turnover.

*Work infrastructure* barriers (*n* = 30) centred on workforce capacity and service availability. Delivering key components, such as assessment or treatment of severe FCR, was seen as dependent on availability of mental health specialists, with access particularly limited in regional centres. Even where mental health specialists were present, demand often exceeded capacity. Enablers (*n* = 35) included using non-mental health specialist staff for screening and triage to address workforce gaps. Extending support into community settings was also proposed, though some raised concerns about GP coordination, long waitlists, and affordability.

*Available resources* barriers (*n* = 31) reflected similar concerns about funding and staffing. Universal or repeated care elements—such as rescreening or booster sessions—were seen as resource-intensive. Some suggested prioritising triage for moderate–high FCR due to constraints. Additional hours for social workers and psychologists were viewed as essential. Equity concerns were raised regarding lack of culturally appropriate measures, limited resources (e.g. care co-ordinators) for some cancer types, and FCR treatment by allied health/psychology, which may not be present in rural/remote areas. Only one response was coded as an enabler, highlighting the tension between need and feasibility.

*Structural characteristics* barriers (*n* = 15) related to organisational processes, such as difficulty capturing all patients at treatment completion and variation across public/private systems. Two-step screening was questioned when referral options were limited: Enablers (*n* = 4) included shared ownership of screening and designated private providers.

*Compatibility* with existing workflows was largely seen as an enabler (*n* = 6). Aligning screening with follow-up appointments was viewed as facilitating timely referral. Administrative barriers (*n* = 3) were noted if FCR screening timing did not align with standard schedules.

#### Outer setting domain

Three enablers, six barriers, and one neutral factor were identified regarding the *Outer Setting*, namely external factors influencing pathway implementation.

*Partnerships and connections *were the main enabler (*n* = 3). Participants highlighted opportunities to deliver FCR interventions outside cancer centres to reduce burden and improve access.

*Local conditions *barriers (*n* = 6) included limited availability of psychological services, long wait times, and costs. Differing cultural attitudes regarding discussing mental health were also noted.

*Financing* for specialist FCR care was questioned. These findings underscore the need for cross-sector partnerships and funding strategies to ensure equitable access.

### Recommended implementation strategies (Aim 3)

**Fig. 1:**
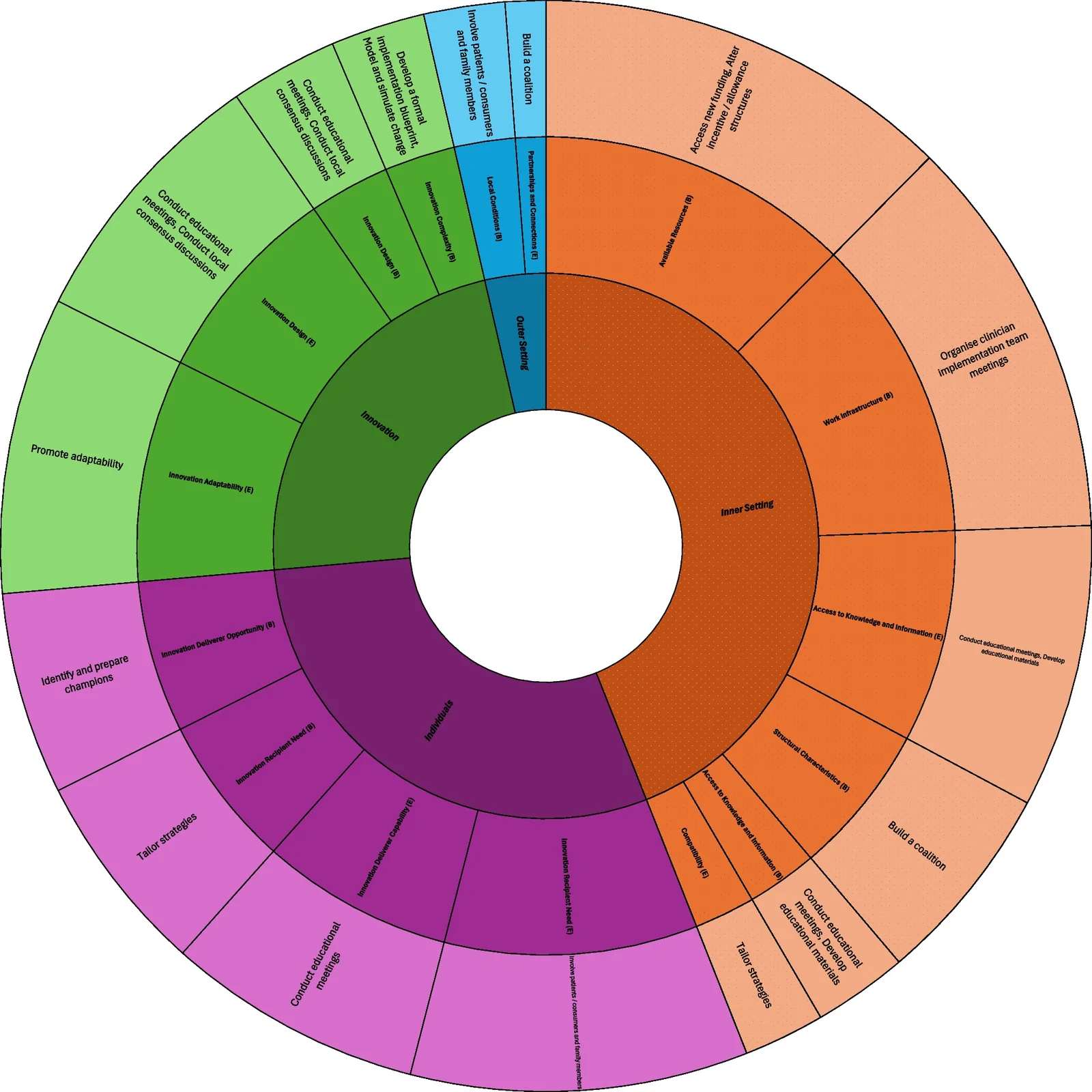
Summary of most the common implementation barriers and enablers by CFIR domain and CFIR-ERIC matched strategies. Legend: The inner ring shows the updated CFIR domain; the middle ring shows the updated CFIR construct, with the number in brackets representing the number of times the construct was coded; the outer ring shows the relevant ERIC implementation strategy

Figure 1 outlines implementation strategies addressing identified barriers and enablers according to the CFIR-ERIC matching tool. Recommended strategies included educational meetings and tailored training resources to foster pathway awareness, ownership, and delivery capability across diverse health professional roles. Emphasis was placed on the pathway’s adaptability and potential for local tailoring to address geographical and resource variability. To overcome limited opportunity among innovation deliverers, preparing local champions to model and support implementation was proposed. Engaging patients and families in shared decision-making was advised to meet varied recipient needs. Organisational strategies—such as building coalitions beyond cancer centres, securing implementation funding, and aligning pathway components with existing workflows—were recommended to support implementation.

## Discussion

This analysis found varied feasibility across FCR clinical pathway elements and multiple implementation barriers and enablers. Triage to stepped care was widely seen as feasible, while recommendations involving universal or repeated screening/intervention posed a feasibility challenge. Key implementation enablers included building capability through training and access to information. Major barriers were resource limitations and structural constraints. Multifaceted implementation strategies, including education provision and adapting pathway delivery to local contexts, may enhance feasibility.

Most participants agreed that pathway elements were feasible, though feasibility ratings were consistently lower than for optimality. Resource-intensive recommendations, such as training all staff in screening, or holding triage conversations with all survivors, were considered least feasible. Feedback underscored tension between what is best for patients and what providers can realistically deliver. Similar concerns were raised regarding implementation of an Australian clinical pathway for anxiety and depression in cancer, with support for structured screening and aftercare, but concerns about resource and role constraints [28]. Prioritising high-impact pathway elements and tailoring to local capacity—such as training selected staff to do screening and focusing triage on those with moderate/severe FCR—may improve feasibility in resource-limited settings.

The pathway design was generally viewed as an enabler, though elements such as two-step screening and delivering universal care before stepping up were seen as adding complexity. While following a single-item FCR screen (e.g. FCR-1r [29]) with a more comprehensive measure (e.g. FCRI-SF [8]) increases administrative burden, it is critical for guiding care based on FCR severity, as per guidelines [18]. Automated electronic patient reported outcome measure (PROM) administration could reduce complexity, and future AI-supported triage may further streamline processes. For now, the second screening tool could be incorporated into triage conversations alongside questions about FCR impact and support needs. Clinician-patient conversations that build on PROMs results can help develop relationships [30] and normalise patients’ FCR [31].

Whether progressive stepped care (starting with universal care) or stratified stepped care (direct allocation to the appropriate level) is preferable likely depends on available resources—another key implementation barrier identified. Progressive stepped care may conserve specialist capacity but risk delaying intensive support, whereas stratified stepped care may improve timeliness but increase costs. Evidence shows minimal outcome differences between approaches in oncology [32], while mental health literature suggests stratified stepped care is more effective but also more costly [33].

Ultimately, while the pathway provides consensus-based, guideline-concordant recommendations, local adaptation is essential. The need for local adaptation to enable equitable access to FCR treatment despite resource constraints has also been identified in Canadian sites implementing the Fear of Recurrence Therapy (FORT) intervention [17]. Developing an implementation blueprint with clear strategies, timelines, and roles, supported by local consensus discussions, is recommended. Process mapping to align new pathways with existing workflows and address context-specific barriers has demonstrated promise in developing implementation plans for other complex aspects of cancer care [34].

Building capability and leveraging existing capacity through training and access to information were key enablers. This aligns with evidence showing lack of accessible training is a major barrier to implementing PROMs in oncology [35]. Recommended strategies include development of training materials and educational sessions. Previous research found 99% of clinical and psychosocial professionals were interested in FCR training [36]. However, training all staff in every aspect of pathway delivery, while ideal, may not be feasible. A modular approach, tailored to roles and resource constraints, may be needed.

Existing FCR training programmes for primary care (in-person) [37] and oncologists (online) [31] have been found to be acceptable and feasible, but programmes for multidisciplinary teams are limited. Face-to-face sessions may improve engagement and visibility, while centralised online resources could support scalability and address staff turnover. Uptake of online training for similar pathways has been low (7%) [38], highlighting the need for organisational support and incentives to encourage participation.

Most pathway implementation barriers pertained to the Inner Setting, largely relating to resources and infrastructure. Staffing, funding, and service limitations—especially in regional and community settings—mirror challenges implementing a cancer clinical pathway for anxiety and depression [28]. These barriers may be mitigated by building capacity across multiple disciplines rather than relying on a single speciality. Identifying local champions is recommended to support uptake, integration, and troubleshooting. Effective champions typically demonstrate influence, ownership, and physical presence at the point of change, combined with persuasiveness, resilience, and a participative leadership style [39]. Building a coalition of implementers is also recommended, and expanding primary care capacity could reduce pressure on tertiary services, particularly in rural areas and for First Nations, ethnic and racial minority populations, who may prefer community-based support [40].

To enhance equitable pathway implementation and reduce health professional burden, coalitions of implementers could include trained lay workers, such as people affected by cancer. With appropriate training, these individuals could assist with patient navigation, help identify those experiencing FCR, connect them with suitable services, and provide emotional support. An umbrella review of patient navigation in cancer care found potential improvements in quality of life and psychological wellbeing, with no clear differences in effectiveness between health professional and lay navigators [41]. Additional funding is critical to enable these strategies, whether for workforce expansion, training, or integration of lay navigators. Investment in FCR care may lower overall healthcare costs [6], making a strong case for prioritising resource allocation for pathway implementation.

Compatibility with existing practices and adaptability to local needs were key implementation enablers. Adaptability has supported PROMs implementation [35] and appears equally important here. Local tailoring—such as defining roles responsible for each pathway element and adjusting referral pathways based on available resources—was also critical to implementing an anxiety and depression clinical pathway [42]. Tailoring to individual needs, including cultural background and health literacy, was strongly endorsed and considered feasible. While co-production of culturally appropriate FCR measures and interventions with communities and end-users is essential [40], collating existing resources for diverse groups could aid tailored pathway implementation in the meantime. Shared decision-making regarding FCR treatment was seen as vital for facilitating engagement, particularly with self-management interventions, consistent with broader survivorship care recommendations [43]. Preferences for FCR support vary [44], and some patients with severe FCR prefer less intensive options [20], despite recommendations for specialist care.

### Strengths and limitations

Study strengths include applying an established implementation determinant framework to a large multidisciplinary dataset and using a recognised taxonomy to map tailored strategies. The updated CFIR was useful for categorising implementation determinants, though some brief responses were difficult to classify. This was mitigated through double-coding and consensus.

While multidisciplinary, the sample was lacking in primary and palliative care clinicians, surgeons, patients, and cultural diversity. Most participants were experienced health professionals working in secondary or tertiary cancer services within Australian public hospitals, so findings may not generalise beyond this context. However, resource constraints appear common across settings [45], suggesting broader applicability of strategies addressing this barrier. Patient perspectives and adaptations for First Nations, ethnic and racial minority populations are being explored in ongoing work.

## Conclusion

Implementing the FCR clinical pathway requires pragmatic adaptation to overcome resource and structural barriers. Resource-intensive recommendations were least feasible, underscoring the need to prioritise high-impact elements, tailor delivery to local contexts and patient needs, and use shared decision-making to optimise engagement. Key implementation strategies include modular training, integration into existing workflows, and use of local champions, supported by implementation coalitions that extend beyond cancer hospitals. Future research should engage stakeholders to contextualise and prioritise strategies and test these in real-world settings using hybrid effectiveness-implementation designs to ensure sustainable delivery and address cancer survivors’ greatest unmet need through routine FCR screening and tailored support.

## Supplementary Information

Below is the link to the electronic supplementary material.

ESM 1 Supplementary Material 1 (DOCX 51.4 KB)

## Data Availability

The datasets generated and analysed during the current study are available from the corresponding author on reasonable request.
