# Supplementary material for: Designing for delivery: a Delphi study of feasibility-informed implementation strategies for a fear of cancer recurrence clinical pathway
Source: Support Care Cancer. 2026 Oct 9;34(11):1079. doi: 10.1007/s00520-026-11275-9 (PMC13652774; doi:10.1007/s00520-026-11275-9)
Supplement: Supplementary file 1 [file 520_2026_11275_MOESM1_ESM.docx]

**Designing for Delivery: A Delphi Study of Feasibility-Informed Implementation Strategies for a Fear of Cancer Recurrence Clinical Pathway**

Supportive Care in Cancer

Authors: Ben Smith^1,2^, Verena Shuwen Wu^1,2^, Alison Pearce^1,3^, Jia Liu^4,5^, Heather L Shepherd^6^, Joanne Shaw^7,8^, Afaf Girgis^2^, Gail Garvey^9^, Laura Kirsten^7,10^, Iman Zakhary^11^, Carolyn Ee^12,13,14^ Dan Ewald^15^, Annie Miller^7^, Andrea Smith^1,3^, Agamjot Kaur Virk^3^, Joy Gao^3^, Natalie Taylor^16^

Affiliations:

1. The Daffodil Centre, The University of Sydney, and Cancer Council NSW, Sydney, Australia
2. South West Sydney Clinical Campuses, UNSW Medicine & Health, UNSW Sydney, Sydney, Australia
3. Sydney School of Public Health, The University of Sydney, Australia
4. The Kinghorn Cancer Centre, St Vincent’s Hospital, Sydney NSW, Australia
5. St Vincent’s Clinical School, UNSW Sydney, Darlinghurst, NSW, Australia
6. Susan Wakil School of Nursing and Midwifery, Faculty of Medicine and Health, The University of Sydney, NSW Australia
7. Psycho-Oncology Cooperative Research Group, The University of Sydney NSW, Australia
8. School of Psychology, Faculty of Science, The University of Sydney, Australia
9. School of Public Health, The University of Queensland, Australia
10. Nepean Cancer Services, Nepean Blue Mountains Local Health District
11. Multicultural Services, Liverpool Hospital, South Western Sydney Local Health District
12. Caring Futures Institute, Flinders University, Australia
13. NICM Health Research Institute, Western Sydney University, NSW, Australia
14. Chris O’Brien Lifehouse Cancer Centre, Camperdown, NSW, Australia
15. Sydney University Medical School, Northern Rivers University Centre for Rural Health, NSW, Australia
16. Implementation to Impact (i2i), School of Population Health, UNSW Sydney, Sydney, Australia

*Corresponding Author*

Ben Smith

The Daffodil Centre, The University of Sydney, and Cancer Council NSW, Sydney, New South Wales, Australia

Email: [ben.a.smith@sydney.edu.au](mailto:ben.a.smith@sydney.edu.au)	Phone: +61 438 634 964

| **Operationalisation of CFIR 2.0 to FCR Clinical Pathway** | | | | |
| --- | --- | --- | --- | --- |
| **Domain** | **Construct** | **General Definition**  **The degree to which:** | **Context Definition**  **The degree to which:** | **Inclusion/Exclusion Notes** |
| **1. INNOVATION DOMAIN**  *The proposed clinical pathway for FCR and its various components (e.g., screening, triage, stepped care, treatment) is the innovation, i.e. the “thing” being implemented.* | | | | |
| **A. Innovation source** | | The group that developed and/or visibly sponsored use of the innovation is reputable, credible and/or trustable. | The group that developed and/or visibly sponsored use of clinical pathway for FCR is reputable, credible and/or trustable. |  |
| **B. Innovation evidence base** | | The innovation has robust evidence supporting its effectiveness. | The proposed clinical pathway for FCR have robust evidence supporting their effectiveness. | *Include* statements regarding awareness of/desire for evidence & quality/validity of evidence. Include statements regarding clinical utility as well as psychosocial, practical utility. |
| **C. Innovation relative advantage*** | | The innovation is better than other available innovations or current practice. | The proposed clinical pathway for FCR are better than other available innovations or current practice. | *Exclude* statements relating to a strong need for clinical pathway for FCR and/or that the current situation is untenable. Code these to: **3E. Tension for Change.** |
| **D. Innovation adaptability*** | | The innovation can be modified, tailored, or refined to fit local context or needs. | The proposed clinical pathway for FCR can be modified, tailored, or refined to fit local context or needs. | E.g. is the pathway suitable for remote communities? |
| **E. Innovation trialability*** | | The innovation can be tested or piloted on a small scale and undone. | The proposed clinical pathway for FCR can be tested or piloted on a small scale and undone. |  |
| **F. Innovation complexity** | | The innovation is complicated, which may be reflected by its scope | The proposed clinical pathway for FCR are more complicated than typical | Include statements about the complexity of the proposed clinical pathway for FCR, not the |

|  | and/or the nature and number of connections and steps. | cancer support, which may be reflected by its screening, triage and stepped treatment. | complexity of implementation of the clinical pathway. |
| --- | --- | --- | --- |
| **G. Innovation design** | The innovation is well designed and packaged, including how it is assembled, bundled, and presented. | The proposed clinical pathway for FCR are well designed and packaged, including how it is assembled, bundled, and presented. | Include statements related to how the clinical pathway for FCR is presented to deliverers and recipients. |
| **H. Innovation cost*** | The innovation purchase and operating costs are affordable. | The medical costs of the proposed clinical pathway for FCR are affordable. | *Exclude* costs related to space, time, or resource trade-offs for implementation of the proposed clinical pathway for FCR. **Instead, code these to: 3. Inner Domain.** |
| 2. **OUTER SETTING DOMAIN**   ***Outer Setting:*** The setting in which the Inner Setting exists, e.g., hospital system, school district, state. There may be multiple Outer Settings and/or multiple levels within the Outer Setting (e.g., community, system, state).   - Broader level – health system (state-level), Australian cancer care system/policies/care plans | | | |
| **A. Critical incidents** | Large-scale and/or unanticipated events disrupt implementation and/or delivery of the innovation. | Large-scale and/or unanticipated events disrupt implementation and/or delivery of professionally led clinical pathway of for FCR. | *Exclude* staff turnover (i.e. of facilitator) unless specifically related to the critical event.  e.g. the pandemic |
| **B. Local attitudes** | Sociocultural values (e.g., shared responsibility in helping recipients) and beliefs (e.g., convictions about the worthiness of recipients) encourage the Outer Setting to support implementation and/or delivery of the innovation. | Sociocultural values (e.g., shared responsibility in helping people with FCR  ) and beliefs (e.g., convictions about the worthiness of people with FCR) encourage the Outer Setting to support implementation and/or delivery of professionally led clinical pathway for  FCR. | E.g. attitudes surrounding mental health or trust in mental health professionals |
| **C. Local conditions** | Economic, environmental, political, and/or technological conditions | Economic, environmental, political, and/or technological conditions enable | Unlikely to have statements coded here – more likely to code to **3. Inner Domain / J.** |

|  | enable the Outer Setting to support implementation and/or delivery of the innovation. | the Outer Setting to support implementation and/or delivery of clinical pathway for FCR. | **Available resources / 1. Funding**). Broader conditions can be included here (such as economic or political conditions not included in E. Policies and laws) |
| --- | --- | --- | --- |
| **D. Partnerships and connections** | The Inner Setting is networked with external entities, including referral networks, academic affiliations, and professional organization networks. | The Inner Setting is networked with external entities, including referral networks, academic affiliations, and professional organization networks. |  |
| **E. Policies and laws** | Legislation, regulations, professional group guidelines and recommendations, or accreditation standards support implementation and/or delivery of the innovation. | Legislation, regulations, professional group guidelines and recommendations, or accreditation standards support implementation and/or delivery of clinical pathway for FCR |  |
| **F. Financing** | Funding from external entities (e.g., grants, reimbursement) is available to implement and/or deliver the  innovation. | Funding from external entities (e.g., grants, reimbursement) is available to implement and/or deliver clinical  pathway for FCR. | Include statements that related to external funding. Such as reimbursement, funding through government, and other external  entities. |
| **G. External pressure** | External pressures drive implementation and/or delivery of the innovation.  *Use this construct to capture themes related to External Pressures that are not included in the subconstructs below.* | External pressures drive implementation and/or delivery of clinical pathway for FCR.  *Use this construct to capture themes related to External Pressures that are not included in the subconstructs below.* |  |
| **1. Societal pressure** | Mass media campaigns, advocacy groups, or social movements or  protests drive implementation and/or delivery of the innovation. | Mass media campaigns, advocacy groups, or social movements or  protests drive implementation and/or delivery of clinical pathway for FCR. |  |
| **2. Market pressure** | Competing with and/or imitating  peer entities drives implementation and/or delivery of the innovation. | Competing with and/or imitating peer  entities drives implementation and/or delivery of clinical pathway for FCR. |  |

| **3. Performance-measurement pressure** | Quality or benchmarking metrics or established service goals drive implementation and/or delivery of the innovation. | Quality or benchmarking metrics or established service goals drive implementation and/or delivery of clinical pathway for FCR. | E.g. healthcare KPIs set by the hospital surrounding psychosocial health outcomes of patients |
| --- | --- | --- | --- |
| 3. **INNER SETTING DOMAIN**   ***Inner Setting:*** The setting in which the innovation is implemented, e.g., hospital, school, city. There may be multiple Inner Settings and/or multiple levels within the Inner Setting, e.g., unit, classroom, team.   - Designed to be delivered in tertiary, hospital-based setting (screening most likely to occur in hospitals) - Treatment component may happen in community-based setting - Cancer services (different centres across tumour streams?) - Capture that there may be different types of enablers/barriers for different settings depending on step/component of the pathway | | | |
| ***Note:*** | *Constructs A – D exist in the Inner Setting regardless of implementation and/or delivery of the innovation, i.e., they are persistent general characteristics of the Inner Setting.* | | |
| **A. Structural characteristics** | Infrastructure components support functional performance of the Inner Setting.  *Use this construct to capture themes related to Structural Characteristics that are not included in the subconstructs below.* | Infrastructure components support functional performance of the Inner Setting (e.g. hospital, cancer centre or community organisation).  *Use this construct to capture themes related to Structural Characteristics that are not included in the subconstructs below.* |  |
| **1. Physical infrastructure** | Layout and configuration of space and other tangible material features support functional performance of the Inner Setting. | Layout and configuration of space and other tangible material features support functional performance of the Inner Setting (e.g. hospital, cancer  centre or community organisation). | Differs from J.  Include statements related to physical infrastructure supporting the general functional performance of inner setting |
| **2. Information technology infrastructure** | Technological systems for  tele-communication, electronic documentation, and data storage, management, reporting, and analysis support functional performance of the Inner Setting. | Technological systems for  tele-communication, electronic documentation, and data storage, management, reporting, and analysis support functional performance of the |  |

|  |  | Inner Setting (e.g. hospital, cancer centre or community organisation). |  |
| --- | --- | --- | --- |
| **3. Work infrastructure** | Organization of tasks and responsibilities within and between individuals and teams, and general staffing levels, support functional performance of the Inner Setting. | Organization of tasks and responsibilities within and between individuals and teams, and general staffing levels, support functional performance of the Inner Setting (e.g. hospital, cancer centre or community organisation). |  |
| **B. Relational connections** | There are high quality formal and informal relationships, networks, and teams within and across Inner Setting boundaries (e.g., structural, professional). | There are high quality formal and informal relationships, networks, and teams within and across Inner Setting (e.g. hospital, cancer centre or community organisation) boundaries (e.g., structural, professional). | This relates to the characteristics of the team  - how team is structured, etc.  (more abstract elements about the structure and organisation of team compared to the concrete way the team communicates and shares information) |
| **C. Communications** | There are high quality formal and informal information sharing practices within and across Inner Setting boundaries (e.g., structural, professional). | There are high quality formal and informal information sharing practices within and across Inner Setting (e.g. hospital, cancer centre or community organisation) boundaries (e.g.,  structural, professional). | Specific elements on how information is shared (formally or informally to ensure coordination of care)  E.g. Good communication between team members to ensure consistent care, a process in place to share medical information |
| **D. Culture** | There are shared values, beliefs, and norms across the Inner Setting. *Use this construct to capture themes related to Culture that are not included in the subconstructs below.* | There are shared values, beliefs, and norms across the Inner Setting (e.g. hospital, cancer centre or community organisation).  *Use this construct to capture themes related to Culture that are not included in the subconstructs below.* |  |
| **1. Human equality-centredness** | There are shared values, beliefs,  and norms about the inherent equal worth and value of all human beings. | There are shared values, beliefs, and  norms about the inherent equal worth and value of all human beings. | Include statements related to the inner  setting’s values being to provide equal and fair treatment |
| **2. Recipient-centredness** | There are shared values,  beliefs, and norms around caring, | There are shared values,  beliefs, and norms around caring, |  |

|  | supporting, and addressing the needs and welfare of recipients. | supporting, and addressing the needs and welfare of people with FCR |  |
| --- | --- | --- | --- |
| **3. Deliverer-centredness** | There are shared values,  beliefs, and norms around caring, supporting, and addressing the needs and welfare of deliverers. | There are shared values,  beliefs, and norms around caring, supporting, and addressing the needs and welfare of deliverers (e.g. counsellors, nurses, social workers). |  |
| **4. Learning-centredness** | There are shared values, beliefs, and norms around psychological safety, continual improvement, and using data to  inform practice. | There are shared values,  beliefs, and norms around psychological safety, continual improvement, and using data to inform practice. |  |
| ***Note:*** | *Constructs E – K are specific to the implementation and/or delivery of the innovation****.*** | | |
| **E. Tension for change** | The current situation is intolerable and needs to change. | The current situation is intolerable and needs to change. |  |
| **F. Compatibility** | The innovation fits with workflows, systems, and processes. | Clinical pathway for FCR fit with workflows, systems, and processes. |  |
| **G. Relative priority** | Implementing and delivering the innovation is important compared  to other initiatives. | Implementing and delivering clinical pathway for FCR is important compared  to other initiatives. |  |
| **H. Incentive systems** | Tangible and/or intangible incentives and rewards and/or disincentives and punishments support implementation and delivery of the innovation. | Tangible and/or intangible incentives and rewards and/or disincentives and punishments support implementation and delivery of clinical pathway for FCR |  |
| **I. Mission alignment** | Implementing and delivering the innovation is in line with the overarching commitment, purpose, or goals in the Inner Setting. | Implementing and delivering clinical pathway for FCR is in line with the overarching commitment, purpose, or goals in the Inner Setting. |  |
| **J. Available resources** | Resources are available to implement and deliver the innovation.  *Use this construct to capture themes related to Available* | Resources are available to implement and deliver clinical pathway for FCR. *Use this construct to capture themes related to Available Resources that are*  *not included in the subconstructs below.* |  |

|  | *Resources that are not included in the subconstructs below.* |  |  |
| --- | --- | --- | --- |
| **1. Funding** | Funding is available to implement and deliver the innovation. | Funding is available to implement and deliver professionally led clinical pathway for FCR. | Costs related to the inner setting - such as funding available to the hospital or community center for the clinical pathway |
| **2. Space** | Physical space is available to implement and deliver the innovation. | Physical space is available to implement and deliver clinical pathway for FCR |  |
| **3. Materials and equipment** | Supplies are available to implement  and deliver the innovation. | Supplies are available to implement and  deliver clinical pathway for FCR. | E.g. IT equipment - telehealth |
| **K. Access to knowledge and information** | Guidance and/or training is accessible to implement and deliver the innovation. | Guidance and/or training is accessible to implement and deliver clinical pathway for FCR | E.g. don’t have access to knowledge/ with additional training |
| 4. **INDIVIDUALS DOMAIN**   *Individuals: The roles and characteristics of individuals.*   - People involved in delivery of clinical pathway (health care professionals from multiple disciplines in hospitals – from inner setting) | | | |
| **ROLES SUBDOMAIN**  ***Project Roles:*** [Document the roles applicable to the project and their location in the Inner or Outer Setting.] | | | |
| **A. High-level leaders** | Individuals with a high level of authority, including key  decision-makers, executive leaders, or directors. | Individuals with a high level of authority, including key  decision-makers, executive leaders in the health system |  |
| **B. Mid-level leaders** | Individuals with a moderate level of authority, including leaders supervised by a high-level leader and who supervise others. | Individuals with a moderate level of authority, including leaders supervised by a high-level leader and who supervise others |  |
| **C. Opinion leaders** | Individuals with informal influence on the attitudes and behaviors of others. | Individuals with **informal** influence on the attitudes and behaviors of others, like researchers. |  |

| **D. Implementation facilitators** | Individuals with subject matter expertise who assist, coach, or support implementation. | Individuals with subject matter expertise who assist, coach, or support implementation of the clinical pathways for FCR |  |
| --- | --- | --- | --- |
| **E. Implementation leads** | Individuals who lead efforts to implement the innovation. | Individuals who lead efforts to implement the delivery of the proposed clinical pathway for FCR. |  |
| **F. Implementation team members** | Individuals who collaborate with and support the Implementation Leads to implement the innovation, ideally including Innovation Deliverers and Recipients. | Individuals who collaborate with and support the Implementation Leads to implement the delivery of clinical pathways for FCR, ideally including Innovation Deliverers and Recipients. |  |
| **G. Other implementation support** | Individuals who support the Implementation Leads and/or Implementation Team Members to  implement the innovation. | Individuals who support the Implementation Leads and/or Implementation Team Members to  delivering the clinical pathway for FCR |  |
| **H. Innovation deliverers** | Individuals who are directly or indirectly delivering the innovation. | Individuals who are directly or indirectly delivering clinical pathway for FCR support. |  |
| **I. Innovation recipients** | Individuals who are directly or  indirectly receiving the innovation. | Individuals who are attending the  clinical pathway of stepped treatment | - if issue from individual's side |
| **CHARACTERISTICS SUBDOMAIN**  ***Project Characteristics:*** [Document the characteristics applicable to the roles in the project based on the COM-B system (Michie 2011) or role-specific theories.]   - Distinguish between **role** and **characteristics** of different people involved (e.g., people delivering screening or treatment) | | | |
| **A. Need** | The individual(s) has deficits related to survival, well-being, or personal fulfillment, which will be addressed by implementation and/or delivery of the innovation. | The individual(s) has deficits related to survival, well-being, or personal fulfillment, which will be addressed by implementation and/or delivery of screening, triaging and treatment groups. |  |
| **B. Capability** | The individual(s) has interpersonal competence, knowledge, and skills to fulfill Role. | The individual(s) has interpersonal competence, knowledge, and skills to | - staff have/don’t have necessary skills |

|  |  | the implementation of screening, triaging and treatment groups. |  |
| --- | --- | --- | --- |
| **C. Opportunity** | The individual(s) has availability, scope, and power to fulfill Role. | The individual(s) has availability, scope, and power to the implementation of screening, triaging and treatment groups. | - anything where the staff may not have the available time to complete the jobs - cluster patient-clinician relationships |
| **D. Motivation** | The individual(s) is committed to fulfilling Role. | The individual(s) is committed to the role of delivering the screening,  triaging and treatment support.. |  |
| 5. **IMPLEMENTATION PROCESS DOMAIN**   ***Implementation Process:*** The activities and strategies used to implement the innovation.  ***Project Implementation Process:*** [Document the implementation process framework (Nilsen 2015) and/or activities and strategies (Powell 2011; Powell 2015) being used to implement the innovation. Distinguish the implementation process used to implement the innovation (activities that end after implementation is complete) from the innovation (the “thing” that continues when implementation is complete) (Curran 2020; Lengnick-Hall 2022; Pinnock 2017).]   - **Potentially not relevant (could look at assessing needs, preferences of deliverers/recipients)** | | | |
| **A. Teaming** | Join together, intentionally coordinating and collaborating on interdependent tasks, to implement the innovation. | Join together, intentionally coordinating and collaborating on interdependent tasks, to implement the proposed clinical pathways for FCR. |  |
| **B. Assessing needs** | Collect information about priorities, preferences, and needs of people. *Use this construct to capture themes related to Assessing Needs that are not included in the*  *subconstructs below.* | Collect information about priorities, preferences, and needs of people.  *Use this construct to capture themes related to Assessing Needs that are not included in the subconstructs below.* |  |
| **1. Innovation deliverers** | Collect information about the priorities, preferences, and needs of deliverers to guide implementation and delivery of the innovation. | Collect information about the priorities, preferences, and needs of deliverers to guide implementation and delivery of the proposed clinical pathways for FCR |  |

| **2. Innovation recipients** | Collect information about the priorities, preferences, and needs of recipients to guide implementation and delivery of the innovation. | Collect information about the priorities, preferences, and needs of recipients to guide implementation and delivery ofthe proposed clinical pathway for FCR |  |
| --- | --- | --- | --- |
| **C. Assessing context** | Collect information to identify and appraise barriers and facilitators to implementation and delivery of the innovation. | Collect information to identify and appraise barriers and facilitators to implementation and delivery of the proposed clinical pathway for FCR |  |
| **D. Planning** | Identify roles and responsibilities, outline specific steps and milestones, and define goals and measures for implementation success in advance. | Identify roles and responsibilities, outline specific steps and milestones, and define goals and measures for implementation success in advance. |  |
| **E. Tailoring strategies** | Choose and operationalize implementation strategies to address barriers, leverage  facilitators, and fit context. | Choose and operationalize implementation strategies to address barriers, leverage facilitators, and fit  context. |  |
| **F. Engaging** | Attract and encourage participation in implementation and/or the innovation.  *Use this construct to capture themes related to Engaging that are not included in the subconstructs below.* | Attract and encourage participation in implementation of the proposed clinical pathway for FCR  *Use this construct to capture themes related to Engaging that are not included in the subconstructs below.* |  |
| **1. Innovation deliverers** | Attract and encourage deliverers to serve on the implementation team and/or to deliver the innovation. | Attract and encourage deliverers to serve on the implementation team and/or to deliver the proposed clinical  pathway for FCR. |  |
| **2. Innovation recipients** | Attract and encourage recipients to serve on the implementation team and/or participate in the innovation. | Attract and encourage people with cancer survivors of FCR to serve on the implementation team and/or participate in the proposed stepped treatment. |  |

| **G. Doing** | Implement in small steps, tests, or cycles of change to trial and cumulatively optimize delivery of the innovation. | Implement in small steps, tests, or cycles of change to trial and cumulatively optimize delivery of the proposed clinical pathway for FCR. |  |
| --- | --- | --- | --- |
| **H. Reflecting and evaluating** | Collect and discuss quantitative and qualitative information about the success of implementation.  *Use this construct to capture themes related to Reflecting & Evaluating that are not included in the subconstructs below.* | Collect and discuss quantitative and qualitative information about the success of delivery of the proposed clinical pathway for FCR  *Use this construct to capture themes related to Reflecting & Evaluating that are not included in the subconstructs below.* |  |
| **1. Implementation** | Collect and discuss quantitative and qualitive information about the success of implementation. | Collect and discuss quantitative and qualitive information about the success of implementation of the proposed  clinical pathway for FCR |  |
| **2. Innovation** | Collect and discuss quantitative and qualitative information about the success of the innovation. | Collect and discuss quantitative and qualitative information about the success of the proposed clinical pathway for FCR. |  |
| **I. Adapting** | Modify the innovation and/or the Inner Setting for optimal fit and integration into work processes. | Modify the pathway for FCR and/or the Inner Setting for optimal fit and integration into work processes. |  |
